# Homelessness and associated factors over a 13-year period among psychiatric in-patients in Berlin, Germany: routine data analysis

**DOI:** 10.1192/bjo.2023.501

**Published:** 2023-06-29

**Authors:** Dario Jalilzadeh Masah, Meryam Schouler-Ocak, Stefan Gutwinski, Kirsten Gehrenbeck, Karl Deutscher, Daniel Schindel, Sonia Lech, Stefanie Schreiter

**Affiliations:** Department of Psychiatry and Neurosciences, Charité Campus Mitte, Charité – Universitätsmedizin Berlin, Berlin, Germany; Institute for Medical Sociology and Rehabilitation Science, Charité – Universitätsmedizin Berlin, Berlin, Germany; Department of Psychiatry and Neurosciences, Charité Campus Mitte, Charité – Universitätsmedizin Berlin, Berlin, Germany; and Institute for Medical Sociology and Rehabilitation Science, Charité – Universitätsmedizin Berlin, Berlin, Germany

**Keywords:** Homelessness, mental illness, healthcare use, psychiatric care, in-patient treatment

## Abstract

**Background:**

Homeless patients in psychiatric hospitals are a scarcely studied and there is lack of knowledge about factors associated with homelessness and in-patient treatment.

**Aims:**

To determine the change over time in the number of homeless psychiatric in-patients and to examine factors associated with homelessness.

**Method:**

Retrospective data analysis of 1205 selected electronic patient files on psychiatric in-patient treatment in a university psychiatric hospital in Berlin, Germany. The rate of patients experiencing homelessness over a 13-year period (2008–2021) and the sociodemographic and clinical factors associated with homelessness are analysed over time.

**Results:**

Our study revealed a 15.1% increase in the rate of homeless psychiatric in-patients over the 13-year period. Of the whole sample, 69.3% people lived in secure private housing, 15.5% were homeless and 15.1% were housed in sociotherapeutic facilities. Homelessness was significantly associated with being male (OR = 1.76 (95% CI 1.12–2.76), born outside of Germany (OR = 2.22, 95% CI 1.47–3.34), lack of out-patient treatment (OR = 5.19, 95% CI 3.35–7.63), psychotic disorders (OR = 2.46, 95% CI 1.16–5.18), reaction to severe stress (OR = 4.19, 95% CI 1.71–10.24), personality disorders (OR = 4.98, 95% CI 1.92–12.91), drug dependency (OR = 3.47, 95% CI 1.5–8.0) and alcohol dependency (OR = 3.57, 95% CI 1.67–7.62).

**Conclusions:**

The psychiatric care system is facing an increasing number of patients in precarious social situations. This should be considered in resource allocation planning in healthcare. Individual solutions for aftercare, along with supported housing, could counteract this trend.

Homelessness is a rising social and healthcare challenge in Western countries.^1,2^ People who are homeless are an often marginalised group experiencing various forms of discrimination, such as social exclusion and a lack of health insurance, income, social support and access to the healthcare system.^1,3,4^ Compared with the non-homeless population, being homeless significantly increases the risk of having mental and physical health problems, thus increasing morbidity and mortality.^2,5–7^ A recent meta-analysis of mental illness among homeless people in Western countries found a mean prevalence of at least one current mental disorder of 76.2%, the most common mental conditions being alcohol use disorder (36.7%), drug use disorder (21.7%) and schizophrenia spectrum disorder (12.4%).^8^ This highly exceeds prevalence rates among the general population.^3^ In a similar meta-analysis on studies from Germany, comparable rates were found (the pooled prevalence of axis I disorders was 77.4%), with a higher burden of substance use disorders, especially alcohol dependence.^9^

It is estimated that the global homeless population accounts for 1% of the total population, with significant variations in the change of rates among countries. For example, a 60% increase in homelessness was observed in Latvia between 2010 and 2018, whereas Australia experienced a 5% increase from 2011 to 2016.^10^ Almost yearly from 2008 to 2018 the estimated number of homeless people in Germany increased, to a total of approximately 678 000.^11,12^ There are a variety of reasons for this global trend, including lack of affordable housing, income inequality, the rising cost of living and government policies.^13,14^

With a growing number of people experiencing social exclusion such as homelessness, the healthcare system is confronted with care challenges often connected with social challenges. Although the adaption of the healthcare system to the needs of people undergoing homelessness in order to establish a continuum of care is still an urgent issue, studies on healthcare system use for in-patient treatment among homeless people are scarce and mainly come from North American countries.^15,16^ A Canadian study of administrative data on admissions over a 5-year period showed that homeless patients on surgical and medical wards remained in hospital longer, resulting in substantially higher costs.^16^ However, homeless patients in psychiatric care incurred higher costs that could not be explained by a prolonged length of stay.^16^ A study assessing healthcare utilisation by 1165 people experiencing homelessness in Canada found a subset of ‘high users’ with frequent emergency department attendance.^15^ An analysis of New York City's public general hospitals during 1992–1993 showed an extension of 36% in duration of treatment for homeless patients compared with non-homeless patients. In addition, 80.6% of homeless people had a diagnosis of substance misuse or mental illness.^17^ Hence, the psychiatric care system can be seen as one sector particularly confronted with providing care for homeless people. Studies on healthcare usage among homeless people in Europe are scarce: a prospective cohort study in five European countries found that homelessness predicted length of stay in different directions depending on the country, with a shorter length of stay in Germany, unlike other countries.^18^ A cross-sectional study of 4885 acute psychiatric admissions in London, UK, showed that residential mobility and homelessness were associated with an increase in length of stay.^19^ According to current data, there are significant differences in the length of stay among various Western countries, with Germany being a particular outlier, having shorter length of stay.^18,19^ A study conducted in 1993–1994 in Mannheim found 31% of psychiatric in-patients to be without a private home.^20^ A study in Berlin (*n* = 72) from 1992 showed 10% of in-patients in a psychiatric ward to be homeless and 12% living in inadequate housing conditions.^21^ In the largest German cross-sectional patient survey from Berlin on the housing situation among people in acute psychiatric care from 2016, 13% of participants reported being homeless.^22^ Another German study found a 14% increase in homeless patients between 2016 and 2019 in a routine data study among psychiatric in-patients in North Rhine–Westphalia.^23^ Homeless people are a socially disadvantaged group that frequently experiences stigma and discrimination when trying to access psychosocial and healthcare services. Homeless individuals’ access to health and social care is hindered by bureaucratic procedures, inflexible operating hours, discrimination and stigma.^24,25^

The current literature is especially lacking longitudinal data on healthcare use by homeless people and its influencing factors. As an increasing number of people experiencing homelessness in the general population in Germany as well as worldwide is observed,^10,26^ little is known about its impact on the healthcare system and changes in healthcare system use. In the current study we extracted the electronic patient files of psychiatric in-patients from one of the largest psychiatric hospitals in the centre of Berlin for the period from 2008 to 2021 to explore their housing situation and potentially associated factors. We hypothesised a significant increase in the rate of homeless patients over that period. Potential clinical and sociodemographic predictors for homelessness among psychiatric in-patients are also explored.

## Method

### Study design and sample

In a retrospective study, data on housing status and other sociodemographic and clinical factors were extracted from the electronical medical records of psychiatric in-patients on 12 specific dates (the first of each month) each year between 2008 and 2021. The patient files come from the Psychiatric University Clinic of the Charité at St Hedwig Hospital, which provides psychiatric care for all inhabitants of the districts Wedding, Moabit and Tiergarten in the centre of Berlin, Germany. This hospital is 1 of 15 hospitals in Berlin providing psychiatric mandatory care and cannot decline treatment of compulsory admissions. Patients from other districts are transferred accordingly. The number of people living in these areas increased by about 15.4% between 31 December 2008 and 31 December 2021, from 244 034 to 281 511 people.^27,28^ In these areas live 163 452 people with a migration background (58% of the 281 511 people living there) and 101 114 people (35.9% of the 281 511 people living there) are foreigners (a migration background is defined as including all those who have immigrated to the current territory of the Federal Republic of Germany with a German passport and all Germans born in Germany with at least one parent who immigrated or was born as a foreign national in Germany; foreigners are people living in Germany without German citizenship).^29^ Especially in Wedding, a high percentage of residents receive social welfare benefits.^30^ The hospital offered in-patient treatment to 135 patients at a time in 2008 and 148 patients in 2021 (9.6% increase) in three general psychiatric wards and four specialised wards (addiction, depression, geriatric psychiatry and ‘Soteria’ (treatment of young people with psychotic disorders)). Patients from day clinics or psychiatric out-patient departments were not included in the analysis.

**Fig. 1:**
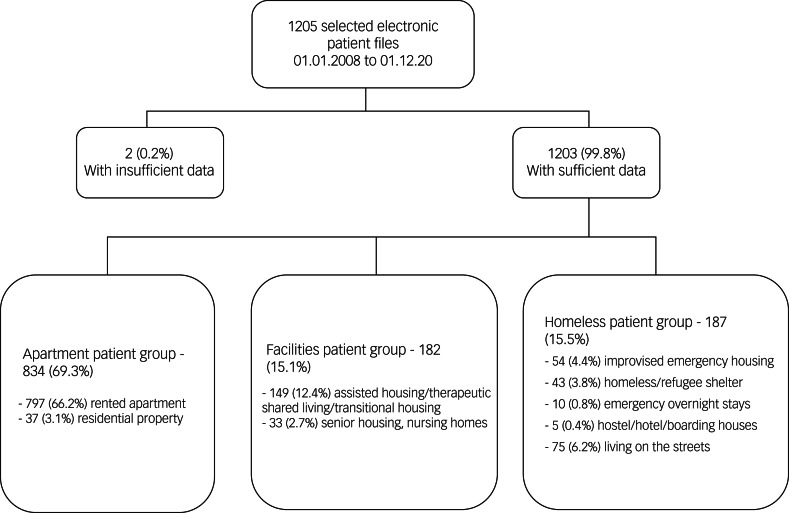
Flow chart showing the clustering process for participants’ housing status.

Numbers of in-patient cases per year varied between 2919 cases in 2015 and 2097 cases in 2012. Patients were selected for data extraction if they were admitted as in-patients on the predefined extraction dates (first of each month). A total of 1205 patients were selected for data extraction on the 156 extraction dates. Two (0.2%) patients were excluded because their accommodation situation before admission could not be determined, leaving a total study sample of 1203 patients. Readmissions counted as new cases since the housing status may have changed during the survey period. In total nine patients have been included twice and two patients have been included three times. A detailed overview of the selection process and distribution of the types of housing can be found in Fig. 1.

### Data extraction

After patient selection, the following sociodemographic and clinical variables were extracted from the electronic patient files into a spreadsheet (Microsoft Excel, Microsoft Office 2019): year of admission, age at admission, gender, country of birth, postcode or ‘no steady address’, and housing status 30 days before admission and directly after discharge. Housing status before admission was categorised into three groups based on a definition by the European Commission:^31^ (a) ‘apartment group’: patients with residential property, a rented apartment or living with family, partner, in parents’ house or a shared apartment with a rental agreement; (b) ‘facilities group’: patients living in assisted housing, therapeutic shared living, transitional housing, senior housing and nursing homes; and (c) ‘homeless group’: patients living in improvised emergency housing conditions, with acquaintances or couch-surfing without a rental agreement, in homeless shelters, emergency overnight stays, hostels, hotels or boarding houses, living on the street, patients directly released from prison without further arrangements or living in refugee shelters.

As clinical variables the following parameters were extracted: psychiatric diagnoses (main and secondary diagnosis), out-patient care (general practitioner, psychiatrist or psychotherapist) in the 6 months prior to admission (yes/no), type of admission (voluntary/compulsory), length of stay, history of suicide attempts (yes/no) and provision of a legal custodian in the 6 months prior to admission (yes/no). The psychiatric diagnoses were recorded based on the discharge diagnoses by the treating physicians, which were based on ICD-10 criteria. For better comparability, the following diagnostic groups were created based on the main groups of ICD-10: organic mental disorders (F0x, F10x.6), psychotic disorders (F2x, F1x.5, F1x.7, F53.1), substance misuse (F1x.1, without F10.1 and F17.1), drug dependence (F1x.2, without F10.2 and F17.2), alcohol misuse (F10.1), alcohol dependence (F10.2), unipolar depression (F32.x, F33.x), bipolar disorders (F31.x), anxiety disorders (F40.x, F41.x), reaction to severe stress (F43.0, F43.1, F43.2), personality disorders (F60.x, F61x F62), intellectual disabilities (F7x.x). Dual diagnosis was defined as the presence of a substance dependence, other than nicotine dependence, and another psychiatric diagnosis.

### Statistical analysis

Further processing and statistical analyses of the data were performed with IBM SPSS version 27.0 for Windows. Standard descriptive analyses with the corresponding statistical parameters (means, s.d.) were calculated depending on the data material. The rates of homeless patients were calculated for each year and the entire study period. We analysed group differences in sociodemographic and clinical variables between housing groups (apartment group, facilities group and homeless group). For the interval-scaled variables, a test for normal distribution was performed using the Shapiro–Wilk test. In the absence of a normal distribution, the Kruskal–Wallis test was used to detect group differences. If significant, the Mann–Whitney test was performed with adjusting for multiple comparisons by using Bonferroni's method. For the analysis of nominal variables, Pearson's chi-squared test was used with a 95% significance level.

A first logistic regression analysis was conducted to examine the association between year of admission and the rate of homelessness. Variables with significant group differences as well as the most frequent diagnoses in the sample of people experiencing homelessness were introduced as factors in a second binary logistic regression model to examine the association between homelessness and sociodemographic and clinical parameters. The variables were examined using Pearson's *r* for correlation before calculating the regression (no significant correlation was found between the variables). Diagnoses with low case numbers were not included in the model. In both models, the presence of homelessness served as the dependent variable (experiencing homelessness: yes/no). Given the exploratory design of this study, no correction for multiple testing was performed.

### Ethical approval

The authors assert that all procedures contributing to this work comply with the ethical standards of the relevant national and institutional committees on human experimentation and with the Helsinki Declaration of 1975, as revised in 2008. All procedures involving human subjects/patients were approved by the local ethics committee of the Charité Berlin (number: EA1/057/20).

## Results

### Sociodemographic background of the sample

The average age of the population was 43.2 years (s.d. = 16.3); 40.2% (*n* = 485) were female. Patients had an average length of stay in the hospital of 22.2 days (range: 1–355 days). As regards country of birth, 76.2% (*n* = 918) were born in Germany; the country of birth could not be determined for 0.3% (*n* = 4) and for the remaining 283 patients, countries of birth were distributed among 46 countries (Turkey 7.8%, Poland 2.5%, Russia 1% and others).

### Housing situation

**Fig. 2:**
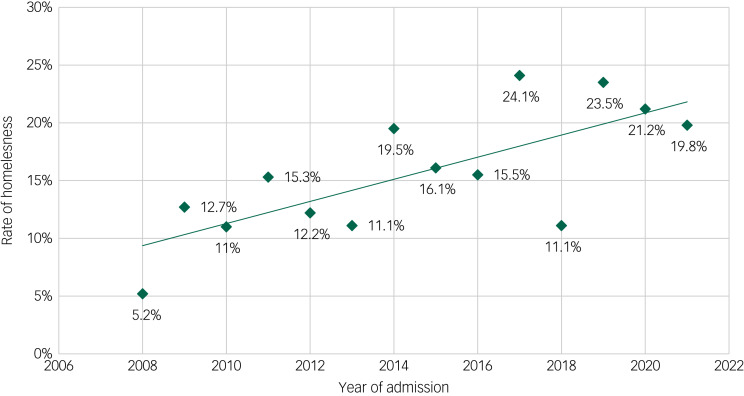
Rates of homelessness among psychiatric in-patients from 2008 to 2021.

Among the total sample of 1203 patients, 69.3% were living in a private apartment or house (apartment group), 15.1% were living in sociotherapeutic facilities (facilities group) and 15.5% were homeless (homeless group). Detailed information on housing group distribution can be found in Fig. 1. The rate of homelessness showed a significant increase over time (5.2% in 2008; 19.8% in 2021; Fig. 2) (regression coefficient 0.077; OR = 1.080, 95% CI 1.039–1.123; *P* = <0.001).

The comparison of the housing groups showed significant group differences in age, gender and country of birth: patients in the homeless group were significantly younger (mean 38 years (s.d. = 11.9), *P* = <0.001), more often male (78.6%, *P* = <0.001) and more often not born in Germany (36.9%, *P* = <0.001) than patients in the apartment group or facilities group (Table 1).

**Table 1:** Demographic and clinical parameters for the three housing groups (*n* = 1203**)**

	Apartment group (*n* = 834)	Facilities group (*n* = 182)	Homeless group (*n* = 187)	Statistics
Demographic characteristics				
Age, years: mean (s.d.)^a^	44.31 (16.7)	43.51 (17.56)	38.02 (11.93)	*H* = 18.01 *P* < 0.001
Proportion of females, *n* (%)^b^	365 (43.8)	79 (43.4)	40 (21.4)	χ² = 32.7 *P* < 0.001
Country of birth Germany, *n* (%)^b^	645 (77.6)	154 (85.1)	118 (63.1)	χ² = 26.651 *P* < 0.001
Healthcare use characteristics				
Length of stay, days: mean (s.d.)^c^	23.31 (31.82)	19.53 (33.82)	20.55 (38.84)	*H* = 17.55 *P* < 0.001
Legal custodian in 6 months prior to admission^b^^,^^d^	234 (28.1)	104 (57.1)	63 (33.7)	χ² = 56.885 *P* < 0.001
Out-patient care in 6 months prior to admission, *n* (%)^b^^,^^e^	488 (69.8)	121 (80.1)	48 (29.1)	χ² = 118.748 *P* < 0.001
Compulsory admission, *n* (%)^b^^,^^f^	86 (10.3)	20 (11)	31 (16.7)	χ² = 6.062 *P* = 0.048
Psychiatric disorders (ICD 10 criteria) and clinical characteristics				
Organic mental disorders, *n* (%)^b^	51 (6.1)	26 (14.3)	5 (2.7)	χ² = 21.684 *P* < 0.001
Psychotic disorders, *n* (%)	242 (29)	60 (33)	57 (30.5)	χ² = 1.157 *P* = 0.561
Alcohol dependence, *n* (%)^b^	142 (17)	26 (14.3)	48 (25.7)	χ² = 9.704 *P* = 0.008
Drug dependence, *n* (%)^b^	77 (9.2)	30 (16.5)	22 (11.8)	χ² = 8.456 *p* = 0.015
Substance misuse, *n* (%)	7 (0.08)	2 (1.1)	0 (0)	χ² = 1.805 *P* = 0.406
Unipolar depression, *n* (%)^b^	115 (13.8)	4 (2.2)	9 (4.8)	χ² = 29.019 *P* = <0.001
Bipolar disorders, *n* (%)	38 (4.6)	4 (2.2)	5 (2.7)	χ² = 3.11 *P* = 0.211
Anxiety disorders, *n* (%)	12 (1.4)	0 (0)	0 (0)	χ² = 5.363 *P* = 0.068
Reaction to severe stress, *n* (%)^b^	70 (8.4)	4 (2.2)	22 (11.8)	χ² = 12.128 *P* = 0.002
Personality disorders, *n* (%)^b^	47 (5.6)	18 (9.9)	18 (9.6)	χ² = 6.774 *P* = 0.034
Intellectual disabilities, *n* (%)^b^	6 (0.7)	8 (4.4)	1 (0.7)	χ² = 17.309 *P* < 0.001
Dual diagnosis, *n* (%)^b^^,^^g^	148 (17.7)	42 (23.1)	46 (24.6)	χ² = 6.177 *P* = 0.046
Suicide attempts in history (yes/no), *n* (%)	99 (12.4)	19 (10.9)	31 (18)	χ² = 4.797 *P* = 0.09

a. Significant group difference after Mann–Whitney test and after adjusted alpha level by Bonferroni's method (α/3; *P* = 0.016) between apartment group and homeless group. No significant group difference between apartment group and facilities group and or between facilities group and homeless group.

b. Significant group difference.

c. Significant group difference after Mann–Whitney test and after adjusted alpha level by Bonferroni's method (α/3; *P* = 0.016) between apartment group and homeless group and between facilities group and homeless group. No significant group difference between apartment group and facilities group.

d. Total *n* = 1147, apartment group *n* = 801, facilities group *n* = 174, homeless group *n* = 172.

e. Total *n* = 1015, apartment group *n* = 699, facilities group *n* = 151, homeless group *n* = 165.

f. Total *n* = 1200, apartment group *n* = 832, facilities group *n* = 182, homeless group *n* = 186.

g. Dual diagnosis was defined as the presence of substance dependence, other than nicotine, and another psychiatric diagnosis.

The housing status of the apartment group after admission changed during the in-patient treatment in 31 (3.8%) cases: 24 (2.8%) were discharged to sociotherapeutic facilities and 7 (0.008%) became homeless (reasons for losing their home: loss of apartment due to eviction, termination of lease contract due to imprisonment). In the facilities group, 5 (2.7%) became homeless after treatment and 1 person got their own apartment. In the homeless group, 14 (7.5%) were discharged to sociotherapeutic facilities and in the subgroup of patients living on the streets, 48 (33.3%) were discharged into (emergency) shelters.

### Distribution of clinical variables

The sample showed significant group differences regarding the distribution of diagnoses. In the facilities group, patients were diagnosed significantly more often with organic mental disorders, intellectual disabilities and drug dependence than in the other groups; unipolar depression was diagnosed significantly more often in the apartment group, alcohol dependence and reaction to severe stress in the homeless group (Table 1). Personality disorders were least frequently diagnosed in the apartment group. There were no significant group differences for schizophrenia/psychotic disorders between the groups. For dual diagnosis, there was a significant group difference, with the highest proportion (24.6%) in the homeless group.

The groups showed significant differences in length of stay, with the apartment group showing the longest stays. Less than one-third (29.1%) of the homeless group had received out-patient care in the 6 months prior to admission, which was significantly lower than in the other groups (*P* = <0.001). The facilities group differed significantly from the other groups regarding a higher rate of legal custodianship (57.1%, *P* = <0.001). Furthermore, the rate of compulsory admission showed a significant group difference, being highest in the homeless group (16.7%, *P* = 0.048).

### Binary logistic regression model

After introducing significant variables and the six most common diagnoses from the group comparisons into a binary logistic regression with rate of homelessness as the dependent variable, we identified being male (OR = 1.7, 95% CI 1.12–2.76; *P* = 0.001), country of birth outside of Germany (OR = 2.2, 95% CI 1.47–3.34; *P* = <0.001), a lack of out-patient care (OR = 5.19, 95% CI 3.35–7.63; *P* = <0.001), presence of a psychotic disorder (OR = 2.46, 95% CI 1.16–5.18; *P* = 0.018), drug dependence (OR = 3.47, 95% CI 1.5–8.0; *P* = 0.004), alcohol dependence (OR = 3.57, 95% CI 1.7–10.2; *P* = 0.001), reaction to severe stress (OR = 4.19, 95% CI 1.71–10.24; *P* = 0.002) and personality disorders (OR = 4.98, 95% CI 1.92–12.91; *P* = 0.001) as factors significantly associated with an increased chance of being homeless (Table 2).

**Table 2:** Predictors of homelessness in the study population (*n* = 1021): multivariable binary logistic regression model

Predictor	Adjusted OR (95% CI)	*P*
Gender (male *v*. female)	1.76 (1.12–2.76)	0.01***
Age	0.99 (0.979–1)	0.35
Country of birth outside Germany	2.22 (1.47–3.34)	<0.001***
Lack of out-patient care	5.19 (3.35–7.63)	<0.001***
No legal custodian	0.88 (0.57–1.36)	0.58
Length of stay	1 (0.99–1)	0.85
Dual diagnosis	0.87 (0.57–1.34)	0.54
Psychotic disorder	2.46 (1.16–5.18)	0.018***
Drug dependence	3.47 (1.50–8.01)	0.004***
Alcohol dependence	3.57 (1.67–7.62)	0.001***
Unipolar depression	1.28 (0.46–3.5)	0.62
Reaction to severe stress	4.19 (1.71–10.24)	0.002***
Personality disorders	4.98 (1.92–12.91)	0.001***

***P < 0.001.

## Discussion

This study is the first longitudinal retrospective analysis of electronic patient files of psychiatric in-patients over a 13-year period for rates of homelessness and associated factors in a European urban area (Berlin, Germany). Over the whole study sample, only 69.3% of our patient sample lived in secure private housing prior to admission, 15.1% were living in sociotherapeutic facilities and the overall rate of homelessness was 15.5%. This rate is clearly higher than the rate of homelessness in the general population in Germany, which is below 1%, and shows that homeless people are overrepresented in the hospital-based psychiatric care system.^32^

### Sociodemographics

Although there has been no change in social assistance in Berlin over the study period, there has been a significant decrease in affordable housing and insufficient construction of new social housing, despite a growing population. It has been reported that these factors are partly responsible for urban homelessness.^33^ Furthermore, our data showed a significant increase (by 15%) in the proportion of homeless patients between 2008 and 2021. This result clearly exceeds the increase in homelessness in Germany's general population over that period, estimated to be 4.4% (excluding homeless refugees, who were only included since 2015 in the estimation model).^11,12^ Although the number of inhabitants in the hospital's catchment area increased by 15.3% between 2008 and 2021, the in-patient capacity of the hospital increased by only 9.6%. An increase in homelessness is not yet considered in the planning of healthcare capacities, potentially resulting in a shift of people with mental disorders and need for in-patient treatment who are living in secure living conditions to out-patient settings or longer waiting times.

The homeless population in this study shows similar demographic characteristics to the homeless population of North Rhine–Westphalia, Germany.^23^ Here, too, patients experiencing homelessness were significantly younger and more often male.^23^ Other factors, such as involuntary admissions and shorter lengths of stay, are also consistent with this study.^23^ Young age is considered a risk factor for homelessness in psychiatric patients according to a Danish study.^34^ An explanation for the younger age of homeless patients is the considerably increased risk of death and association with high mortality, as shown by Nilsson et al.^35^ Another meta-analysis from high-income countries shows that homeless individuals are more likely to suffer from severe somatic illnesses, which lead to lower life expectancy.^36^ Since our data do not provide information about first onset of the disorder or the last date of accommodation, we cannot say whether homelessness preceded the onset of mental illness or the mental illness caused the loss of housing.

### Predictors of homelessness

In our regression model, being male, being born outside of Germany, the lack of out-patient treatment prior to admission, as well as the diagnoses of psychotic disorders, drug and alcohol dependence, reaction to severe stress and personality disorders, were identified as factors significantly associated with being homeless.

Being male was significantly associated with being homeless, which is in line with other study samples reporting high rates of males among homeless people.^37,38^ Males are more likely to be affected by substance misuse, psychotic disorders and involuntary treatment and are less likely to access social services and support systems, and as a result often remain untreated for longer periods.^37,39^ The proportion of homeless females (21.4%) is consistent with national estimates for 2018 (25%),^11^ but both estimates may be inaccurately low owing to hidden homelessness among females. Homeless females more often avoid social and medical support provided for homeless people, since they report a feeling of insecurity in, for example, male-dominated homeless shelters or due to previous traumatic experiences, which should be considered by the psychiatric care system.^40,41^ Overall, it could be considered that homeless people of different genders may experience homelessness differently and have different needs, which implies the need for gender-specific support services.

In our sample, the probability of being homeless was two times higher for people born outside of Germany than for people born in Germany. People who have had traumatic experiences in their lives were up to four times more likely to be homeless. There is a link between migration and stress-related disorders, with refugees suffering more often from post-traumatic stress disorder.^42^ Migrants may have lower language skills, limited access to safe housing, healthcare and income support and ongoing family insecurities, which can enhance stress-related reactions and might contribute to the risk of homelessness.^42^ Furthermore, during their first months in Germany, refugees are often housed in shelters falling under the definitions of homelessness and struggle to find regular housing after.

Among the six most common psychiatric diagnoses, reaction to severe stress and personality disorders showed a strong link in the regression model with being homeless. The link between reaction to severe stress and homelessness has been described, showing that homeless people often report some form of abuse^43^ and are at higher risk of experiencing traumatic events during homelessness.^44^ A meta-analysis from 2021 has shown that the lifetime prevalence of adverse childhood experiences (ACEs) is substantially higher among people experiencing homelessness in the USA and Canada than in the general population and exposure to ACEs might be associated with prevalence of mental illness, substance misuse and victimisation.^45^ In addition, another meta-analysis showed that personality disorders are common among homeless people in Western countries,^46^ with an increased risk of dropping out of treatment.^47^

In our sample, the lack of out-patient treatment increased the probability of homelessness by up to five times. In a Canadian study based on longitudinal data on healthcare utilisation by homeless people and their age- and gender-matched low-income controls in a universal health insurance system, homeless people had substantially higher rates of emergency department and hospital use than the general population controls, especially driven by a subset of high users.^15^ Out-patient care often comes with barriers for homeless people (inflexibility of appointment scheduling, lack of health insurance causing refusal of treatment by doctors, bureaucratic procedures). In our sample, only 29.1% of the homeless group were having any kind of medical out-patient treatment in the 6 months prior to admission, which is less than in a survey among homeless individuals from Hamburg (47% did not have any out-patient treatment in the 3 months prior).^48^

According to a recent meta-analysis among Western countries, psychotic disorders are highly prevalent among homeless people, with pooled prevalence rates for schizophrenia spectrum disorders of 12.4% (95% CI 9.5–15.7%).^8^ In our sample, having a psychotic disorder was significantly associated with being homeless. In cities, the factor of urbanicity seems to be a risk factor for psychotic disorders by itself.^30^ A recent US study underlined the importance of minimising the risk of homelessness by early treatment and detection of psychotic disorder, which was linked to youth-onset homelessness.^49^

Drug and alcohol dependence were significantly associated with homelessness in our sample. A US study showed alcohol and drug dependence to be associated with first-time homelessness, underlining the importance of early detection, destigmatisation and treatment of substance dependence in preventing homelessness.^50^ Lack of effective aftercare for patients with substance use disorders coupled with being discharged back into homelessness can lead to a high risk of relapse.^51^

The significantly high proportion of people with a drug use disorder living in sociotherapeutic facilities is surprising, since abstinence is usually required and is often a barrier for many homeless people with substance use. On the other hand, the high rates of substance use among homeless people in our sample indicates the need for sociotherapeutic facilities with lower thresholds regarding substance consumption.

### Barriers to treatment and length of stay

Some institutional processes, such as waiting lists and telephone enrolment in drug and alcohol treatment programmes, establish barriers for people without housing to receive psychiatric treatment. The shorter length of stay for homeless people compared with privately housed people in our sample supports findings from a study comparing length of stay for psychiatric in-patients among five European countries.^18^ In Germany, homelessness predicted a shorter length of stay, in contrast to a longer length of stay in most other countries.^49^ These results could reflect an established system of support for homeless people in Berlin outside of the hospital setting (e.g. charitable medical support, substance use support programmes), but might also reflect difficulties of supporting homeless people during psychiatric in-patient treatment efficiently, resulting in treatment drop-out. In combination with the finding of significantly higher rates of compulsory admissions among homeless patients in our sample, compulsory treatment might lead patients to keep their stay as short as possible and get discharged as soon as the legal basis for a compulsory admission has run out, increasing mistrust in institutions.

### Limitations

There are several limitations to our study that must be outlined. Routine data can be prone to errors. The generalisability of our results to other areas, especially rural areas, is limited since our study region represents an urban district. Another limitation to generalisability is the exception of German studies showing a shorter length of stay for homeless patients compared with studies in other European and North American countries. Still, the catchment area of this study is characterised by a high proportion of European migrants and relatively low socioeconomic status and is thus comparable to other European urban areas. Furthermore, the study sample was limited to an in-patient psychiatric treatment setting and did not include day clinics, the emergency room and out-patient centres of the hospital. Future research should expand the homelessness sample, by including homeless people from different in- and out-patient healthcare services. We did not include data for housing status after treatment, insurance status or form of discharge, such as ‘against medical advice’, owing to absence of standardised documentation. Since Germany has a mandatory universal healthcare system, numbers of uninsured people are low. Nevertheless, approximately 61 000 people (less than 1%) in Germany remain without healthcare insurance.^52^

Finally, owing to the cross-sectional design we cannot draw causal relationships between homelessness and associated factors, such as gender, place of birth or type of mental disorder. Future research should include longitudinal designs when examining protective and risk factors of homelessness among people with mental disorders.

## Data Availability

Since the analyses are based on routine data from electronic patient files, original data cannot be shared.
