# Homelessness and associated factors over a 13-year period among psychiatric inpatients in Berlin, Germany – A routine data analysis – CORRIGENDUM

**DOI:** 10.1192/bjo.2023.538

**Published:** 2023-08-01

**Authors:** Dario Jalilzadeh Masah, Meryam Schouler-Ocak, Stefan Gutwinski, Kirsten Gehrenbeck, Karl Deutscher, Daniel Schindel, Sonia Lech, Stefanie Schreiter

This article was originally published with author Kirsten Gehrenbeck's name spelled incorrectly. The error has been corrected.
